# Microglia cannibalism during neurodevelopment results in necroptotic cell death

**DOI:** 10.1371/journal.pbio.3002869

**Published:** 2024-10-31

**Authors:** F. Chris Bennett, Mariko L. Bennett

**Affiliations:** 1 Department of Psychiatry, Perelman School of Medicine, University of Pennsylvania, Philadelphia, Pennsylvania, United States of America; 2 Division of Neurology, Children’s Hospital of Philadelphia, Philadelphia, Pennsylvania, United States of America; 3 Department of Neurology, Perelman School of Medicine, University of Pennsylvania, Philadelphia, Pennsylvania, United States of America

## Abstract

During brain development, neurons are selectively cleared by microglia to sculpt neural circuits. This primer discusses a recent study in PLOS Biology showing that microglia also clear away debris from other microglia, which is followed by microglial necroptosis.

## Teaser

Clearance of dying neurons by microglia is critical to healthy neurodevelopment, but what else do microglia eat? A new study in *PLOS Biology* demonstrates that microglia not only eat neurons but each other, and that this “microglia cannibalism” causes necroptotic cell death.

**Fig 1:**
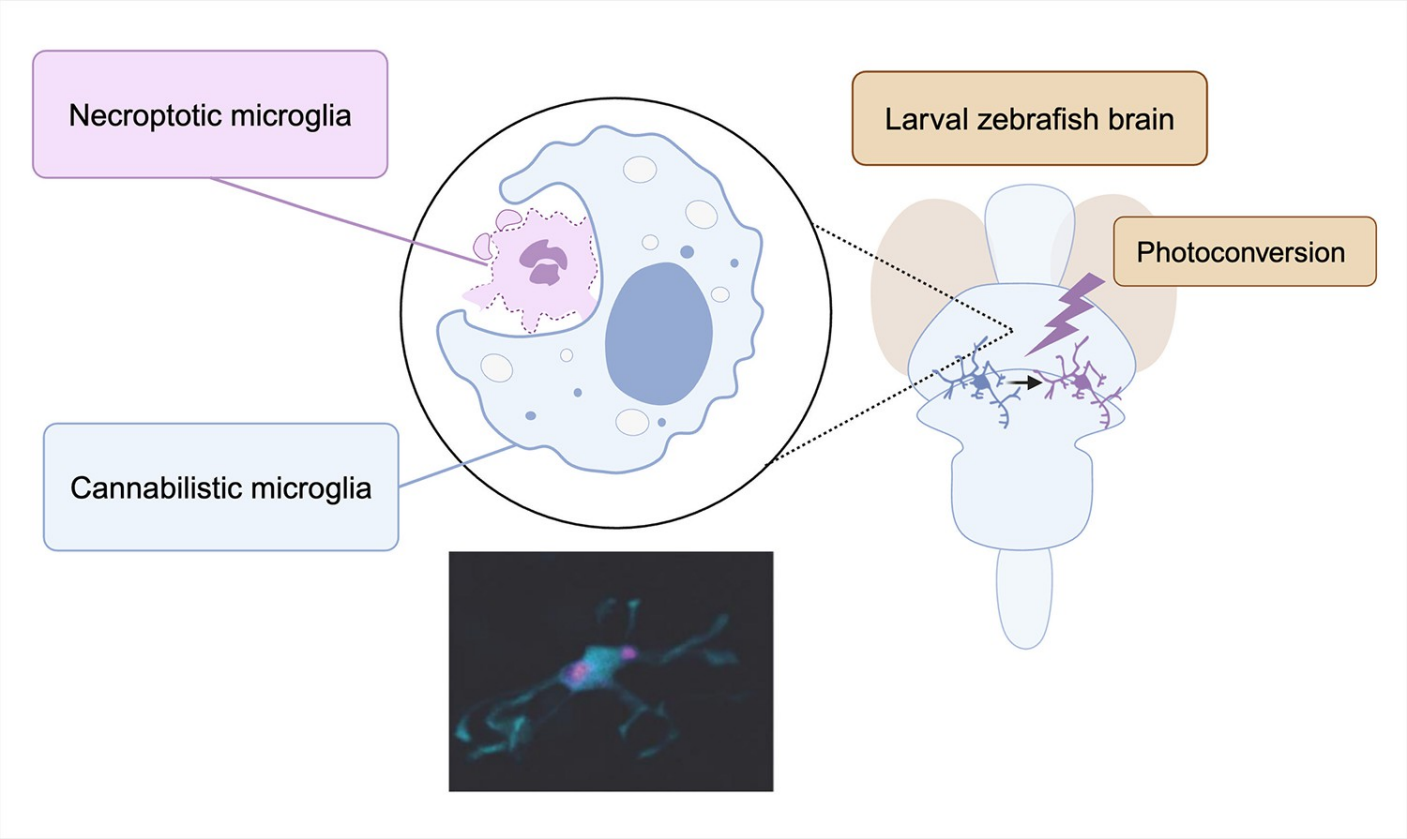
Efferocytic microglia are cannibalized by other microglia. Microglia are responsible for clearing apoptotic cells during normal development, which causes them to undergo necroptosis. This leads to clearance by other microglial cells, which ultimately die. Created with Biorender.com.

Though it has had myriad interpretations since the 14th century BCE, the Ouroboros (the serpent eating its own tail) is thought to symbolize renewal and the circle of life. In contrast, the adage “a serpent must eat a serpent to become a dragon,” suggests potentially nefarious or aggrandizing consequences [1]. But what of the fate of cells that eat other cells? Critical to neural circuit development is overproduction. More neurons, axons, and synapses are made than constitute the mature brain, and this feature—overexpansion followed by selective clearance—leads to careful sculpting of neural circuits. Microglia, brain parenchymal macrophages, are the central effectors of apoptotic neuron clearance [2]. As professional phagocytes, microglia are specialized to detect and digest dying neurons without disrupting the fragile homeostasis of the nascent central nervous system (CNS). In a landmark study, Gordon and colleagues report a dramatic new discovery: during early embryonic development, microglia eat not only neurons but the corpses of other microglia, and when they do, they die by necroptosis, an inflammatory form of cell death [3] (Fig 1).

Using a zebrafish model with fluorescently tagged microglia, the authors deeply explored a simple observation: In the days after fertilization, when microglia progenitors start to colonize the developing CNS and proliferate, some of them shrink up and burst, leaving behind debris that is rapidly engulfed and removed by neighboring microglia. The authors were able to blunt this phenomenon with small molecule inhibitors of necroptosis, an inflammatory death pathway where cellular contents leak into the extracellular space. Although inhibition of apoptosis, a more controlled and immunoquiescent cell death mechanism also reduced microglial death, time-lapse imaging and staining for apoptotic markers suggested that nearly all microglial death was necroptotic. To validate, the authors expressed a photoconvertable fluorophore in microglia, allowing them to use laser light to selectively label microglia with high spatiotemporal control. This approach allowed the authors to verify the surprising finding that many intact microglia disappear during development, after which their debris appears in phagocytic vacuolar structures of other microglia, which then themselves die. In addition, microglia also undergo necroptosis after efferocytosis of other non-microglial cells. It remains unclear why and which microglia undergo cannibalism after debris clearance and the mechanisms underlying necroptosis after cannibalism, which can be addressed in follow-up studies. But in sum, the authors paint a clear picture of a cycle, where microglia “cannibalize” necroptotic microglial debris of their neighbors, inducing their own subsequent necroptotic death.

The existence of microglia-dependent microglial necroptosis raises many exciting scientific questions. Colonization of the developing brain by microglia, a topic of great interest, is mostly framed as a process of infiltration, proliferation, and migration. Gordon, Schafer, and Smith argue that the regulation of microglia numbers is more complex, and also includes mechanisms to actively contract the microglial pool, adding an additional layer of regulation during tissue development. It is intriguing that microglial death arises through necroptosis, a process considered highly inflammatory and important for strong antiviral responses in infection. Why would the CNS rely on an inflammatory and self-propagating mode of death, when inflammation is commonly associated with tissue damage? One hypothesis is that while the rate of macrophage phagocytosis is carefully regulated by lysosomal flux [4], perhaps even under normal circumstances, there must be a “cannibalism safety net,” to prevent promiscuous phagocytosis of important structures. In addition, there is increasing awareness that immune effector molecules, including traditional “inflammatory” mediators, are instrumental to proper CNS formation and function, including “eat me” signals [5], “find me” signals [6], regulators of cell state [7], and programmed death regulation [8]. It is tempting to speculate that in addition to controlling microglial numbers, cannibalism may serve other roles across these categories.

Future studies will clarify the generalizability of microglia cannibalism, but there is a good chance that the work of Gordon and colleagues is important in both the developing and adult brain. As one important example, in a chemical model of demyelination, microglial necroptosis was critical for successful remyelination [9]. Inflammatory microglial death is also already implicated in the pathogenesis of Alzheimer disease. In adulthood, microglia typically tile the brain, but in AD, plaque-associated microglia are phagocytic, receive innate immune signals, and are in close proximity to each other. Furthermore, amyloid exposure is already known to cause pyroptotic cell death [10], and microglia cannibalism would not be surprising in this context. Since pyroptosis spurs plaque formation, elucidating the molecular mechanisms of microglial necroptosis holds potential to identify new therapeutic targets across the lifespan. Outside the brain, there is evidence that macrophage necroptosis plays an important physiological role. After Listeria monocytogenes infection, Kupffer cells, the tissue-resident macrophages of the liver, die by necroptosis [11]. This recruits “replacement” Kupffer cells by hepatocyte IL-33 signaling, and protective anti-bacterial responses, suggesting a mechanism for necroptosis in maintenance of cell numbers and tissue repair. Overall, this work provides evidence—for the first time—that microglia cannibalize other microglia and that eating other cells results in necroptotic cell death. Whether this process represents the sustainability of microglia (the Ouroboros) or dangerous microglia (the dragon) remains a topic for future debate.
